# Mechanisms of High-Grade Serous Carcinogenesis in the Fallopian Tube and Ovary: Current Hypotheses, Etiologic Factors, and Molecular Alterations

**DOI:** 10.3390/ijms22094409

**Published:** 2021-04-23

**Authors:** Isao Otsuka

**Affiliations:** Kameda Medical Center, Department of Obstetrics and Gynecology, Kamogawa 296-8602, Japan; otsuka.isao@kameda.jp

**Keywords:** ovarian cancer, high-grade serous carcinoma, carcinogenesis, molecular alterations

## Abstract

Ovarian high-grade serous carcinomas (HGSCs) are a heterogeneous group of diseases. They include fallopian-tube-epithelium (FTE)-derived and ovarian-surface-epithelium (OSE)-derived tumors. The risk/protective factors suggest that the etiology of HGSCs is multifactorial. Inflammation caused by ovulation and retrograde bleeding may play a major role. HGSCs are among the most genetically altered cancers, and *TP53* mutations are ubiquitous. Key driving events other than *TP53* mutations include homologous recombination (HR) deficiency, such as BRCA 1/2 dysfunction, and activation of the CCNE1 pathway. HR deficiency and the *CCNE1* amplification appear to be mutually exclusive. Intratumor heterogeneity resulting from genomic instability can be observed at the early stage of tumorigenesis. In this review, I discuss current carcinogenic hypotheses, sites of origin, etiologic factors, and molecular alterations of HGSCs.

## 1. Introduction

Ovarian cancer is the most lethal gynecological malignancy. Epithelial ovarian cancers (EOCs) are a heterogeneous group of diseases and can be divided into five main types, based on histopathology and molecular genetics [1]: high-grade serous, low-grade serous, endometrioid, clear cell, and mucinous tumors. These tumors may be classified into type I and II tumors. Type I tumors include endometriosis-related tumors (endometrioid and clear cell carcinomas), low-grade serous carcinoma, and mucinous carcinoma. Type II tumors are composed of high-grade serous carcinomas, for the most part [2]. Although this classification conflicts with recent molecular insights into the etiology of EOCs [3], type II tumors that also include carcinosarcomas could be classed together.

High-grade serous carcinoma (HGSC) is the most common and lethal subtype of EOC, as most women with HGSC are diagnosed at a late stage, when achieving a cure is rare [4]. The vast majority of serous carcinomas are high-grade tumors [5]. To develop an effective method for prevention and early detection, elucidation of carcinogenesis is essential. Recently, our understanding of the origins and pathogenesis of HGSC has substantially progressed through whole genome and bioinformatic analyses. This review discusses the current carcinogenic hypotheses, sites of origin, etiologic factors, and molecular alterations of HGSCs.

## 2. Carcinogenic Hypotheses and Risk/Protective Factors

### 2.1. Incessant Ovulation

**Figure 1:**
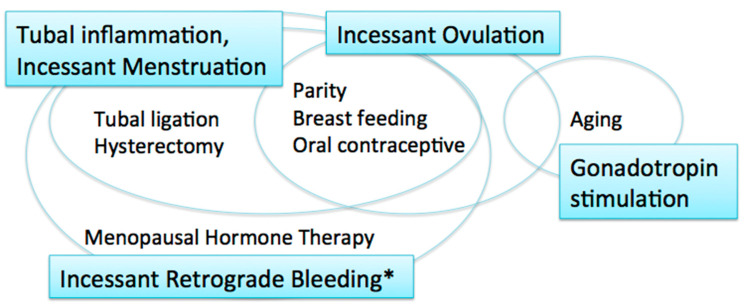
Carcinogenic hypotheses and risk/protective factors. * including menstruation and postmenopausal bleeding.

EOCs were traditionally thought to arise from the ovarian surface epithelium (OSE). The OSE is the pelvic mesothelium that overlies the ovary and lines ovarian epithelial inclusion cysts, and is derived from the coelomic epithelium [6,7]. The risk/protective factors for EOCs include parity, breast-feeding, and oral contraceptive use; all of these factors reduce ovarian cancer risk [8] (Figure 1). These reproductive and hormonal factors are associated with ovulation suppression. Thus, the risk of EOC is thought to be associated with the number of ovulatory cycles.

The incessant ovulation hypothesis was proposed by Fathalla in 1971 based on these observations [9]. This hypothesis proposes that recurrent damage and repair of the OSE from repeated ovulation increase the risk of cell damage and subsequent neoplastic transformation. A study showed that a higher number of ovulatory cycles may be associated with increased amounts of DNA damage [10].

### 2.2. Gonadotropin Stimulation

The majority of women with ovarian cancer present in the postmenopausal period, when pituitary gonadotropin levels are elevated. The gonadotropin stimulation hypothesis proposes that high gonadotropin levels can have an effect on OSE cells and promote carcinogenesis [11,12]. Gonadotropins that persist in high levels for many years after menopause may stimulate the OSE, in which gonadotropin receptors are expressed, and OSE cells may subsequently undergo malignant transformation [12]. Surges of gonadotropins that initiate each ovulation may also play a role in carcinogenesis; thus, the incessant ovulation and gonadotropin hypotheses are interrelated.

### 2.3. Tubal Inflammation

These two hypotheses, however, have limitations. If the number of lifetime ovulatory cycles and exposure to high levels of gonadotropins are associated with EOC development, fertility treatment might increase the risk of EOC via the multiple ovulations stimulated by gonadotropins, specifically luteinizing hormone (LH) and follicle stimulating hormone (FSH). In fact, studies suggest that there is no significant relationship between in vitro fertilization treatment using gonadotropin stimulation and subsequent risk of EOC [13,14]. In addition, as the ovulatory rupture sites appear to be random, the repeated rupture and repair that occurs with each ovulation should not affect the same population of surface epithelial cells [15]. Furthermore, neither the incessant ovulation nor gonadotropin stimulation hypotheses explains the protective effect of tubal ligation and hysterectomy on the development of EOC [16].

The fallopian tube, in particular the fimbriae, emerged as another site of origin based on findings related to prophylactic surgery for ovarian cancer risk reduction in women with genetic predisposition to the disease [17]. Based on these observations, the tubal inflammation hypothesis was proposed by Salvador [7]. Chronic inflammation is known to be a risk for cancer. The fallopian tube is regularly exposed to a variety of inflammatory agents, and can show signs of acute and chronic inflammation, through the process of retrograde bleeding from the endometrial cavity during menstruation. Infection also induces inflammation in the fallopian tube [7]. Chlamydia trachomatis infection may be associated with serous carcinogenesis [18].

### 2.4. Incessant Menstruation

The incessant menstruation hypothesis, proposed by Vercellini [16], also explains why ovarian cancer risk is decreased by tubal ligation and ovulation suppression. In this theory, pathogenesis of endometriosis-associated carcinomas, specifically endometrioid and clear cell carcinomas, as well as serous carcinomas, can be explained. In serous carcinomas, retrograde menstruation from the endometrial cavity into the Douglas pouch is the causative mechanism that generates fallopian tube inflammation [7,16]. The incessant menstruation hypothesis explains ovarian cancer risk well in the premenopausal period. However, in postmenopausal women, another risk factor is related to ovarian carcinogenesis, that is, menopausal hormone therapy (MHT).

### 2.5. Incessant Retrograde Bleeding

Incessant retrograde bleeding is an expansion of the concept of incessant menstruation and may explain more accurately the etiology of HGSCs in both premenopausal and postmenopausal women [19]. Some types of MHT, also called hormone replacement therapy, increase ovarian cancer risk. The risk of serous ovarian cancer differs by regimen of MHT. Its risk in women with intact uteri is increased with the use of estrogen alone, and estrogen with sequentially added progestin [20,21]. Both regimens cause endometrial bleeding. In contrast, serous ovarian cancer risk is not altered by the use of continuous estrogen and progestin, which results in endometrial atrophy with bleeding cessation [19,21]. Thus, MHT regimens that cause endometrial bleeding are associated with an increased risk of serous ovarian carcinoma.

## 3. Sites of Origin of HGSCs

Although the dominant site of origin for HGSCs is the distal fallopian tube (fimbriae) [17,22], HGSCs can also arise from the ovary [23,24,25,26,27]. In experimental models, HGSCs developed from both the fallopian tube and ovary with inactivation of a few genes [26,28,29,30]. Cancer may originate from the transition of stem cells, as the acquisition of multiple mutagenic events can occur in long-lived stem cells that are capable of self-renewal [31], and stem cells can be found both in the fallopian tube and ovary, in particular in the distal fallopian tube and in the transition area between the OSE, mesothelium, and tubal epithelium [23,32,33].

### 3.1. Fallopian Tube Epithelium

A substantial percentage (60–88%) of HGSCs originate in the fallopian tube [17,22,25,34,35], both in women with *BRCA* mutations and in sporadic cases [2,17,36]. The molecular profile and immunophenotype of HGSCs are more closely related to the FTE than OSE [22,37,38]. Although fallopian tube epithelial secretory cells are believed to give rise to HGSCs [28], ciliated cells may be another cell-of-origin in the fallopian tube epithelium (FTE) [26].

In the FTE, p53 signatures and serous tubal intraepithelial carcinoma (STIC), both of which are intraepithelial lesions associated with HGSC, can be identified. The p53 signature is a focus of strong p53 immunostaining in benign tubal mucosa [39] and harbors *TP53* mutations and evidence of DNA damage [40]. Telomere shortening occurs in p53 signatures, suggesting that the p53 signature is the earliest precancer lesion [41]. However, the p53 signature may not always be a preneoplastic lesion of HGSC, as p53 overexpression has been found to be common both in *BRCA* carriers and in noncarriers who underwent surgery for benign disease or RRSO [42]. STIC, which is composed of secretory cells showing significant atypia, architectural alterations, a high proliferative index, and strong p53 immunostaining [43], is a putative precursor lesion of HGSC. Identical somatic *TP53* mutations have been detected in the majority of pairs of STIC and concurrent HGSCs [44]. However, not all HGSCs may arise from STIC lesions, even in high-risk women [45]. STIC was observed only in 11–61% (mean 31%) of HGSCs [46], and some STICs are actually metastases from HGSCs, rather than HGSC precursors [47]. Additionally, a subset of serous tubal intraepithelial neoplasias, including STIC, are an intraepithelial metastasis from a contralateral serous tubal intraepithelial neoplasia [48]. STICs and serous tubal intraepithelial lesions (STILs), which are intermediate lesions between the p53 signature and STIC, may not share the protective factors that are associated with HGSC [49]. The development of STICs may be related to random mutations occurring in target cancer drivers, including *TP53* [49]. Thus, many potential precursor or premalignant lesions do not advance to malignant tumors or lethal malignancies [45].

### 3.2. Ovarian Surface Epithelium

The OSE is another site of origin for HGSCs. Ovarian carcinoma in situ has been identified in ovaries removed in risk-reducing oophorectomies in women with a germline *BRCA* mutation [50]. A literature review of microscopic ovarian, fallopian tube, and peritoneal tumors in *BRCA1/2* mutation carriers showed that 60.5% were confined to the fallopian tube only, whereas 21.1% and 2.6% involved only the ovary and only the peritoneum, respectively [35]. Ovarian epithelial inclusion cysts are considered to be a possible site of origin of HGSCs. HGSCs may frequently arise within epithelial inclusion cysts, but not the surface epithelium itself [51]. A dysplastic precursor lesion within epithelial inclusion cysts, showing accumulation of p53, precedes carcinoma development. Recently, the concept of precursor escape has been postulated. Cells from early precursors, such as early serous proliferations, are shed from the fallopian tube and undergo subsequent malignant transformation on the surface of the ovary and peritoneum [52]. There are two known types of ovarian inclusion cysts; one is positive for PAX8 (mullerian marker), and the other is positive for calretinin (mesothelial marker) [53]. However, they may not represent FTE-derived and OSE-derived cysts, as many PAX8-positive cells arise from metaplasia of OSE-derived inclusion cysts [54]. In a mouse model, ectopic tubal-type epithelium (endosalpingiosis) in the ovary did not likely arise as a consequence of detachment and implantation of the tubal epithelium [55].

FTE-derived and OSE-derived HGSCs are different in their pattern of metastasis, transcriptome, and response to chemotherapy [26]. FTE-derived tumors have a greater propensity to disseminate, whereas OSE-derived tumors form large, solitary lesions, with less frequent metastasis [26]. OSE-derived HGSC may have a long latent period and a poor prognosis compared to FTE-derived HGSC [24,25]. The poorer prognosis associated with OSE-derived HGSC may partly be explained by its mesenchymal characteristics. OSE cells have a dual epithelia–mesenchymal phenotype [56], and in the mouse ovaries leiomyosarcoma developed with inactivation of *Brca1* and *Trp53* [57]. A histological subtype showing mesenchymal characteristics had the lowest overall survival among four histological subtypes of HGSC [58].

## 4. Etiologic Factors

**Figure 2:**
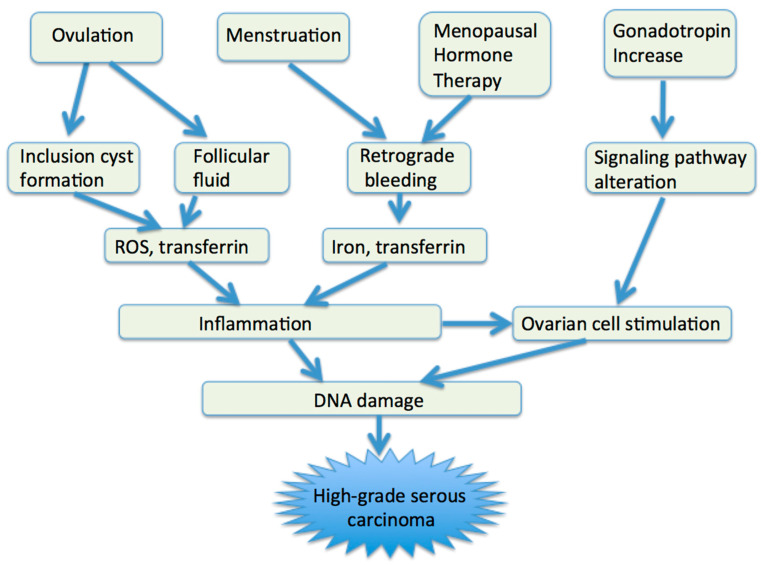
Etiologic factors of HGSCs. ROS, reactive oxygen species.

The etiology of HGSCs appears to be multifactorial (Figure 2). Inflammation, which is induced by follicular fluid released at ovulation and by blood from the endometrial cavity, may play a major role in high-grade serous carcinogenesis [7,16,59,60,61,62,63]. At sites of inflammation, epithelial cells are exposed to high levels of inflammatory mediators such as reactive oxygen species (ROS), cytokines, and growth factors, which contribute to cell proliferation, genetic and epigenetic changes, and cancer development [64].

### 4.1. Ovulation

Ovulation may be associated with high-grade serous carcinogenesis via two aspects: follicular fluid release and inclusion cyst formation. Ovulation is an acute inflammatory process [59], and follicular fluid released from ovulation bathes the fimbrial epithelium and OSE [60]. ROS contained in follicular fluid induce inflammation and DNA double-strand breaks, leading to apoptosis, but if apoptotic failure occurs, neoplastic transformation in fimbrial and ovarian epithelial cells may occur [60,61,62,65]. Exposure of the FTE to follicular fluid can lead to activation of the NF-κB-miR-155 axis, which may represent a possible link between inflammation and DNA damage [62]. Insulin growth factor axis proteins in the follicular fluid confer stemness activation and clonal expansion [63]. Progesterone may prevent ovarian cancer by eliminating p53-deficient epithelial cells [65].

Incessant ovulation may increase the risk of EOC by increasing the risk of inclusion cyst formation. After ovulation, the OSE may invaginate to form clefts and inclusion cysts. Entrapment of exfoliated FTE cells or OSE cells may be an initial event in ovarian carcinogenesis. The estrogen-rich ovarian stromal microenvironment constantly stimulates these cells to proliferate and may cause malignant proliferation. The use of oral contraceptive pills prevents the development of cortical inclusion cysts lined by tubal type epithelium [66]. 

### 4.2. Retrograde Bleeding

Retrograde bleeding from the endometrial cavity into the Douglas pouch, a normal phenomenon during menstrual periods, and subsequent iron-induced oxidative stress, is a causative mechanism of HGSC. The FTE and OSE are bathed in the blood from the endometrial cavity and exposed to the action of catalytic iron and the genotoxic effect of ROS [16]. Iron is an essential nutrient that facilitates cell proliferation and growth, but it also can contribute to tumor initiation and growth. In concert with ROS, transferrin and ferryl hemoglobin, both of which are contained in blood and follicular fluid, may contribute to high-grade serous carcinogenesis [67,68]. Transferrin induces DNA double-strand breaks in murine FTE that may lead to genome instability [67]. Ferryl hemoglobin could rescue p53-deficient fimbrial epithelial cells from lethal ROS stress by consuming extracellular ROS and reducing NADPH oxidase-mediated cell death [68]. Oxidative stress caused by ROS can activate a variety of transcription factors, such as NF-κB, p53, and Wnt/β-catenin, and lead to chronic inflammation [69,70]. Menstrual cytokines, such as tumor necrosis factor (TNF) α and interleukin 8, also cause inflammation [7]. 

### 4.3. Gonadotropin

Gonadotropins—in particular, FSH—may be involved in high-grade serous carcinogenesis. FSH receptor is present in the majority of ovarian epithelial inclusion cysts and ovarian epithelial tumors, suggesting that FSH is an important ovarian epithelial cell growth-promoting factor [71]. FSH promotes proliferation and prevents apoptosis of ovarian cancer cells by activating survivin [72] and supports tumor growth by inducing increased expression of vascular endothelial growth factor (VEGF) [73]. In postmenopausal women, increased FSH levels may foster an inflammatory environment that cannot cause ovulation but may increase ovarian cancer risk through remodeling or morphological changes in the surface epithelium [74]. FSH may alter certain signaling pathways and gene expressions, and result in enhanced proliferation and invasion [12,75].

## 5. Molecular Alterations

**Figure 3:**
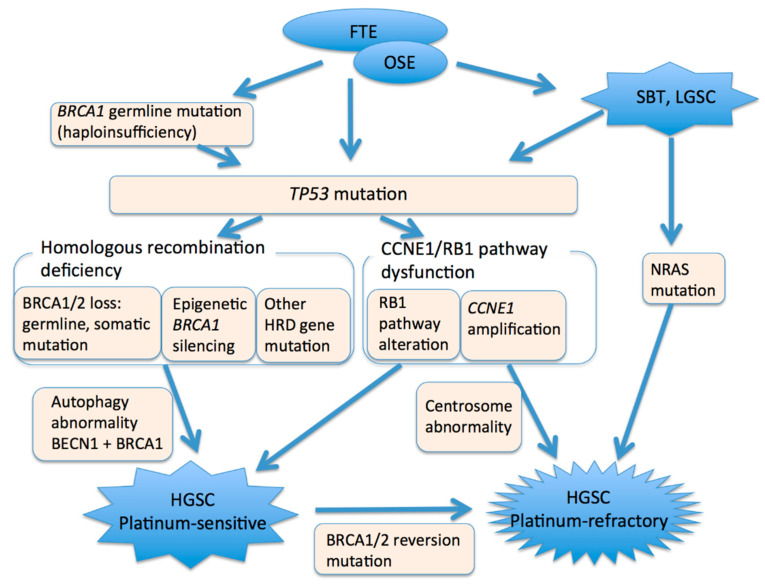
A model of high-grade serous carcinogenesis. FTE, fallopian tube epithelium. OSE, ovarian surface epithelium. SBT, serous borderline tumor. LGSC, low-grade serous carcinoma. HGSC, high-grade serous carcinoma.

HGSCs are among the most genetically altered cancers and are characterized by a few driver mutations and a large number of somatic copy number alterations [76,77]. Driver gene mutations result in inactivation of tumor suppressors and copy number amplifications, leading to an increase in oncogene activity. *TP53* mutations are ubiquitous in HGSCs [78,79,80]. Additional genetic alterations in HGSCs include alterations in the homologous recombination (HR) pathway and alterations in the Rb pathway, or alterations in genes involved in Rb-mediated DNA repair and cell cycle control (Figure 3). HR pathway defects, including *BRCA1/2* dysfunction, appear to be present in at least 50% of these cancers, and alterations in the Rb cell cycle regulation pathway, including *CCNE1* and *RB1* dysfunction, are observed in about 30% [79,81]. Notably, inactivation of *BRCA1* and *BRCA2* is mutually exclusive of amplification of *CCNE1* and inactivation of *RB1* [81,82]. HGSCs can acquire genomic instability via alterations to either pathway [82]. Nearly half of HGSCs have no oncogenic mutations other than *TP53* [83].

### 5.1. TP53

Somatic mutation of *TP53*, which encodes the tumor suppressor p53, is a driver mutation in high-grade serous carcinogenesis. In studies, *TP53* mutations have been identified in 96% of HGSCs [78,79], and are the earliest events in high-grade serous carcinogenesis [84,85]. p53 is responsive to many stress signals and orchestrates diverse cell responses to maintain and restore cell/tissue functions [86]. In response to cellular stresses, such as DNA damage, p53 restrains inappropriate cellular proliferation by triggering transient cell cycle arrest, permanent cell cycle arrest (cellular senescence), and apoptosis; all of these are processes associated with tumor suppression. p53 stimulates various DNA repair mechanisms [87], and its deficiency may permit multiple mutational processes to evolve simultaneously and can enhance cancer initiation [88]. Tumors that lack p53 are commonly characterized by more malignant characteristics, such as poor differentiation and genetic instability [87].

*TP53* mutations in ovarian cancer arise due to spontaneous errors in DNA synthesis and repair, rather than the direct effect of carcinogens [89,90]. During tumor development, a *TP53* mutation is typically followed by loss of heterozygosity, which results in complete p53 deficiency [87]. However, loss of p53 function alone does not produce the malignant phenotype, and at least one more genotoxic event, such as *BRCA1/2* inactivation, is necessary [40].

*TP53* mutations are heterogeneous and occur at almost every codon in the DNA-binding domain of the gene [91]. While loss of function of p53 promotes tumorigenesis, *TP53* mutations may also lead to the development of gain of function (oncomorphic) p53 proteins, which also promote tumorigenesis [91,92]. Oncomorphic *TP53* mutations have been found to be present in 21.3% of ovarian cancers [91].

### 5.2. BRCA1/2

BRCA1/2 dysfunction through germline or somatic mutations of *BRCA1/2*, or epigenetic silencing of *BRCA1* by promoter hypermethylation, is involved in the development of HGSCs [79]. Germline or somatic mutations in *BRCA1/2* have been observed in 20% of cases, and 11% lost *BRCA1* expression through epigenetic silencing. Germline or somatic *BRCA1/2* mutations are mutually exclusive of epigenetic silencing of *BRCA1* [79]. *BRCA1* mutation is distinct from *BRCA2* mutation in several aspects. *BRCA1* mutations are more common than *BRCA2* mutations in HGSCs. Pathogenic germline *BRCA1* mutations, but not *BRCA2* mutations, are more common in younger patients [93]. The cumulative risk of developing ovarian cancer by age 80 years is 44% for *BRCA1* and 17% for *BRCA2* carriers [94]. *BRCA1* mutations are almost exclusively associated with female breast and ovarian cancer, whereas *BRCA2* families are also at risk for male breast cancer, pancreatic cancer in both males and females, and prostate cancers [95]. In EOCs, germline *BRCA1* and *BRCA2* mutations are exclusively associated with high-grade serous histology, and 25% of ovarian HGSCs develop in women with these mutations [96].

*BRCA1* plays a key role in the maintenance of genomic integrity, which is an essential component of its tumor-suppressing function [97]. *BRCA1* is critical in double-strand break repair, utilizing HR. Biallelic inactivation of *BRCA1* is embryonic lethal in mice and, similarly, it is thought to result in cellular lethality in human cells [98]. Tumors with loss of *BRCA1* function require additional somatic mutations, such as in *TP53*, to suppress induction of DNA damage cell cycle checkpoints and escape cell cycle arrest or apoptosis caused by genomic instability [99]. Of note, heterozygous *BRCA1* inactivation results in genomic instability in nontumorigenic breast epithelial cells [98], and heterozygous mutations in *BRCA1* and *BRCA2* have been shown to contribute to development of HGSC in an ovarian cancer mouse model [100]. Downregulation of BRCA1 to levels similar to those present in women with *BRCA1* mutation results in overcoming of the spindle assembly checkpoint [101]. These observations suggest that haploinsufficiency (loss of only one allele) of *BRCA1* may accelerate cancer initiation in women with germline *BRCA1* mutations by facilitating additional genetic alterations [98]. *TP53* mutation, which is caused by *BRCA1* haploinsufficiency, may be associated with the early occurrence of HGSCs in women with germline *BRCA1* mutations.

Not all tumors associated with germline *BRCA1* and *BRCA2* mutations show a loss of BRCA1 or BRCA2 function. Retention of the normal *BRCA1* or *BRCA2* allele is observed in 7% and 16% of *BRCA1* and *BRCA2* germline mutation-associated ovarian cancers, and it is associated with decreased overall survival in patients treated with platinum chemotherapy [99].

*BRCA2*-mutated cases, but not *BRCA1*-mutated cases, indicate a mutator phenotype that contains significantly more mutations [95]. The proteins encoded by *BRCA1* and *BRCA2* work in a common pathway of genomic protection. However, the two proteins work at different stages in the DNA damage response and in DNA repair, and their specific roles are different [102]. As BRCA1 is a pleiotropic DNA damage response protein, its role in DNA repair is broader than that of BRCA2. BRCA1 functions in both checkpoint activation and DNA repair, whereas BRCA2 is a mediator of the core mechanism of HR [102]. *BRCA2*-mutated HGSCs are clinically distinct from *BRCA1*-mutated HGSCs [103]. *BRCA2* mutations may be associated with improved survival compared with either *BRCA* wild-type or a *BRCA1* mutations in ovarian HGSCs [95,103].

HR DNA repair defects may be present in approximately half of all ovarian HGSC cases [79,104]. Biallelic alterations of HR genes such as *BRCA1*, *BRCA2*, *ATM*, *BRIP1*, and *RAD51D* are mutually exclusive of each other in ovarian HGSCs [105].

### 5.3. CCNE1 and Rb1

Amplification of *CCNE1*, which encodes cyclin E1, is a primary oncogenic driver in a subset of HGSCs. *CCNE1* and *RB1* are components of the Rb cell cycle regulation pathway. Cyclin E1 binds to cyclin-dependent kinase 2 (CDK2) and plays an important role in cell cycle progression and in centrosome duplication, which is a strictly regulated process that maintains genetic stability [106]. Amplification of the *CCNE1* copy number occurs early in tumor progression and precedes centrosome amplification [106]. *CCNE1* amplification and *RB1* deletion accelerate the cell cycle, resulting in defective S phase progression and increased chromosome breakage [107]. More than two centrosomes in a cell promote aberrant centrosome duplication and result in chromosomal instability after cytokinesis [106]. *CCNE1* amplification and *RB1* dysfunction are observed in 20% and 10% of HGSCs, respectively [79]. *CCNE1* amplification is more frequent in older women [93] and is associated with a poor prognosis [108]. 

### 5.4. Autophagy Gene

HGSC is the most severely disrupted in terms of autophagy and in compensatory proteostasis pathways among 21 cancer types [83]. Autophagy is an intracellular catabolic degradative process targeting damaged and superfluous cellular proteins, organelles, and other cytoplasmic components [109], and contributes to cell homeostasis and survival [110]. Autophagy is involved in cancer initiation and cancer (stem) cell maintenance [111,112]. In the cancer microenvironment, autophagy can have two functions. In stromal cells, it contributes to tumorigenesis by generating and supplying nutrients to cancerous cells, whereas in immune cells, it may help to support anticancer immune responses [112]. Haploinsufficiency of an autophagy gene, *BECN1*, which is almost always codeleted with *BRCA1*, may permit tumor initiation and potentiate genomic instability in ovarian cancer [76].

### 5.5. Stem Cell Markers

Stem cell markers may be linked to high-grade serous carcinogenesis. Stem cells at the ovarian hilum in mice express a stem cell marker, aldehyde dehydrogenase isoform 1A1 (ALDH1A1), and its loss of expression is an early event in HGSC development [113]. *SOX2* (sex-determining region Y-box2), which is a key stem cell differentiation gene and is required to maintain cancer stem cells [114], is involved in regulation of cancer stem cells, and SOX2 overexpression may occur earlier than *TP53* mutation [115]. 

### 5.6. Transition from Low-Grade Serous Carcinoma

HGSCs may arise from serous borderline tumors and low-grade serous carcinoma. *NRAS* mutation and secondary *TP53* mutation are oncogenic drivers associated with progression from low-grade tumors to high-grade tumors [116,117]. In addition, HGSCs closely associated with serous borderline tumors and low-grade serous carcinoma that lack a *TP53* mutation have been reported [118]. 

## 6. Clinical Relevance

To prevent HGSC development, suppression of the inflammation caused by ovulation and retrograde bleeding may be effective, as well as surgical risk reduction, such as RRSO. Combined OC (estrogen plus progestin) use reduces ovarian cancer risk in women with a *BRCA* mutation [119]. Extended and continuous regimens of combined OC use may be more effective than monthly OCs, which are associated with monthly bleeding. Anti-inflammatory drug use during a bleeding period may reduce ovarian cancer risk [120]. 

Screening for ovarian HGSC needs to detect precursors of FTE-derived and OSE-derived HGSCs. The p53 signature may take a long time (20 years or more) to develop into STIC, whereas STIC may progress to invasive carcinoma in 6–7 years [121,122,123]. For FTE-derived HGSCs, the detection of exfoliated cells and tumor DNA from samples obtained from the endometrial cavity or the cervix may be useful, as precancer or cancer cells in the fallopian tube flow into the endometrial cavity [19,124]. OSE-derived HGSC can be detected early using transvaginal ultrasonography in asymptomatic women aged ≥50 years, or in women aged ≥25 years with a family history of ovarian cancer [125]. SOX2 overexpression, not p53 overexpression, may be used as a molecular precursor for early detection of HGSCs in the fallopian tube [115]. Whereas p53 overexpression (p53 signature) only involves a limited number of cells, SOX2 overexpression is broadly expressed in the non-neoplastic FTE of patients with HGSCs, and also in the normal FTE of *BRCA1* or *BRCA2* mutation carriers who are at high risk for HGSCs.

The mechanisms underlying carcinogenesis are highly associated with treatment response. HR deficient tumors—in particular, *BRCA* dysfunction—are sensitive to platinum-based chemotherapy and PARP inhibitors [104,126,127]. In contrast, HR proficient tumors, such as tumors with *CCNE1* amplification, exhibit primary resistance to these therapies [128,129] and have a poor prognosis [108]. Tumors with *BRCA1/2* dysfunction also respond to immune checkpoint inhibitors [130,131]. In advanced tumors, pretreatment tumor biopsy can be used to predict whether primary complete cytoreductive surgery can be performed [132].

## 7. Concluding Remarks

HGSCs are a heterogeneous group of diseases, and distinct in their site of origin and oncogenic mechanisms [79,133]. Inflammation in the FTE and OSE, caused by ovulation and retrograde bleeding, appears to be associated with their carcinogenesis. Extensive genomic instability, a characteristic of HGSCs, is caused by *TP53* mutation, HR deficiency, and chromosomal instability. Intratumor heterogeneity resulting from genomic instability can be observed at the early stage of tumorigenesis [84,122]. Subclonal tumor populations are present in pretreatment biopsies [134], and recurrent and platinum-resistant tumors develop from pre-existing minor clones [135]. Extensive intratumor heterogeneity limits the effectiveness of targeted therapy, as well as that of chemotherapy, since the targeting of somatic events in all cancer cells is necessary for targeted therapies to be effective [136]. Combinations of targeted therapeutic strategies for multiple clonal or subclonal events may be effective, but their economic and toxicity costs may limit the use of these approaches [136].

The existence of extensive intratumor heterogeneity in HGSCs supports the progression and ultimate lethality of the disease [137]. Therefore, the disease needs to be detected at an early stage, when complete resection of the tumor, which is most associated with long-term survival [138], can be performed. To develop effective methods for prevention and early detection of HGSCs, further studies are needed to identify risk factors separately by site of origin and carcinogenic pathway.
